# Evaluating the effects of laboratory protocols on eDNA detection probability for an endangered freshwater fish

**DOI:** 10.1002/ece3.2083

**Published:** 2016-03-17

**Authors:** Maxine P. Piggott

**Affiliations:** ^1^ Division of EcologyEvolution & GeneticsResearch School of BiologyThe Australian National University44 Daley Rd Acton ACT2601Australia

**Keywords:** Detection probability, environmental DNA, occupancy modeling, PCR

## Abstract

The effectiveness and accuracy of detection using environmental DNA (eDNA) is dependent on understanding the influence laboratory methods such as DNA extraction and PCR strategies have on detection probability. Ideally choice of sampling and extraction method will maximize eDNA yield and detection probability. Determining the survey effort required to reach a satisfactory detection probability (via increased PCR replicates or more sampling) could compensate for a lower eDNA yield if the sampling and extraction method has other advantages for a study, species or system. I analysed the effect of three different sampling and extraction methods on eDNA yield, detection probability and PCR replication for detecting the endangered freshwater fish *Macquaria australasica* from water samples. The impact of eDNA concentration, PCR strategy, target amplicon size and two marker regions: 12S (a mitochondrial gene) and 18S (a nuclear gene) was also assessed. The choice of sampling and extraction method and PCR strategy, rather than amplicon size and marker region, had the biggest effect on detection probability and PCR replication. The PCR replication effort required to achieve a detection probability of 0.95, ranged from 2 to 6 PCR replicates depending on the laboratory method used. As all methods yielded eDNA from which *M. australasica* was detected using the three target amplicons, differences in eDNA yield and detection probability between the three methods could be mitigated by determining the appropriate PCR replication effort. Evaluating the effect sampling and extraction methods will have on the detection probability and determining the laboratory protocols and PCR replication required to maximize detection and minimize false positives and negatives is a useful first step for eDNA occupancy studies.

## Introduction

Determining the presence, distribution and abundance of species is an important tool for monitoring populations and ecological communities. Monitoring surveys often use presence and/or absence data to infer occupancy of a species at a site. Ideally, occupancy surveys would be perfect, such that a species will always be detected if present, and not detected if absent. However, species can go undetected, and failure to account for imperfect detectability in surveys can result in biased estimates of abundance or species richness, impaired detection of change and increase the risk of extinction of rare and endangered species (Wintle et al. 2012). This has led to the development of methods to estimate occupancy rates and correct for imperfect detection probability (MacKenzie et al. 2002; Wintle et al. 2005). These methods use data on the rate of detection and nondetection from multiple surveys, assuming that true occupancy does not vary among surveys, to estimate the probability of detecting a species, given that it is present at a site. Estimating species detectability and survey effort required to accurately infer presence or absence of a species will reduce the impact of false presence and/or absence on monitoring outcomes (Wintle et al. 2005). Thus, an extension of occupancy modeling approaches has been the assessment of survey effort required to reach a satisfactory detection probability for a monitoring program (Wintle et al. 2005; Garrard et al. 2015).

The collection and analysis of aqueous macrobial environmental DNA (eDNA) is showing great promise for improving the detection of freshwater species and overall biodiversity in freshwater and marine environments (Lodge et al. 2012; Taberlet et al. 2012; Thomsen et al. 2012; Pilliod et al. 2013; Bohmann et al. 2014). Studies have successfully detected freshwater fish (Jerde et al. 2011; Lodge et al. 2012; Turner et al. 2014b), amphibians (Dejean et al. 2012; Pilliod et al. 2013), invertebrates (Goldberg et al. 2013; Deiner and Altermatt 2014) and mammals (Foote et al. 2012; Thomsen et al. 2012). Detection of aquatic species using eDNA is dependent on the accuracy of presence and absence data generated from very low starting material and laboratory processes subject to stochastic pressures (Ficetola et al. 2015). Combined with the issue that detections of species are imperfect, it is probable that species can remain undetected using eDNA methods during surveys despite being present.

Occupancy methods are ideally suited to eDNA occupancy surveys, where the probability of detecting a species from a single environmental sample is imperfect, as multiple water samples, DNA extractions or PCR replicates can be used to improve the chance of successfully detecting the target species (Schmidt et al. 2013). Scientists can improve detection probability by increasing survey effort (e.g. samples, extraction or PCR replicates), and this will involve a trade‐off between the costs and risks of false positives and false negatives, financial cost and logistical feasibility (Ficetola et al. 2015; Garrard et al. 2015). Thus, it is important to understand what factors associated with eDNA research influence detection probability. This includes some factors out of the control of scientists, such as aquatic DNA concentration or water chemistry, but it may also be influenced by the choice of methods used for water sampling, DNA extraction and PCR. As discussed in Schmidt et al. (2013), the reliability of a study is likely to improve with additional water samples rather than additional PCR. However, it is important to understand how the choice of eDNA capture and extraction method and PCR strategy influences detection probability, and how to account for this variation in designing the laboratory component of eDNA occupancy surveys.

A large range of different sampling and DNA extraction methods have been used to isolate aqueous macrobial eDNA. Broadly they can be summarized as precipitation followed by extraction with a commercial kit (e.g. Ficetola et al. 2008; Dejean et al. 2011; Thomsen et al. 2012; Piaggio et al. 2014), filtration followed by extraction with a commercial kit (e.g., Goldberg et al. 2011; Jerde et al. 2011; Wilcox et al. 2013) and filtration followed by a Phenol‐Chloroform Isoamyl DNA extraction (e.g., Renshaw et al. 2014; Turner et al. 2014a). However, within these broad groupings, the amount of water, filter type and size, and extraction method vary greatly.

To date, there is no single method that seems applicable to all species, studies and systems. Ideally prior to embarking on an eDNA detection study a range of methods would be tested and optimized and what is considered the “best” method used for field surveys. Determining the “best” method is likely to be based on maximizing the quantity of eDNA but could also be based on ease of sampling. Collecting larger amounts of water is likely to result in greater eDNA concentrations whilst smaller amounts of water may be more suitable for multiple sampling, or remote fieldwork. Determining the survey effort required to reach a satisfactory detection probability (via increased PCR replicates or more sampling) could compensate for a lower eDNA yield if the sampling and extraction method has other advantages for a study, species or system.

**Figure 1:**
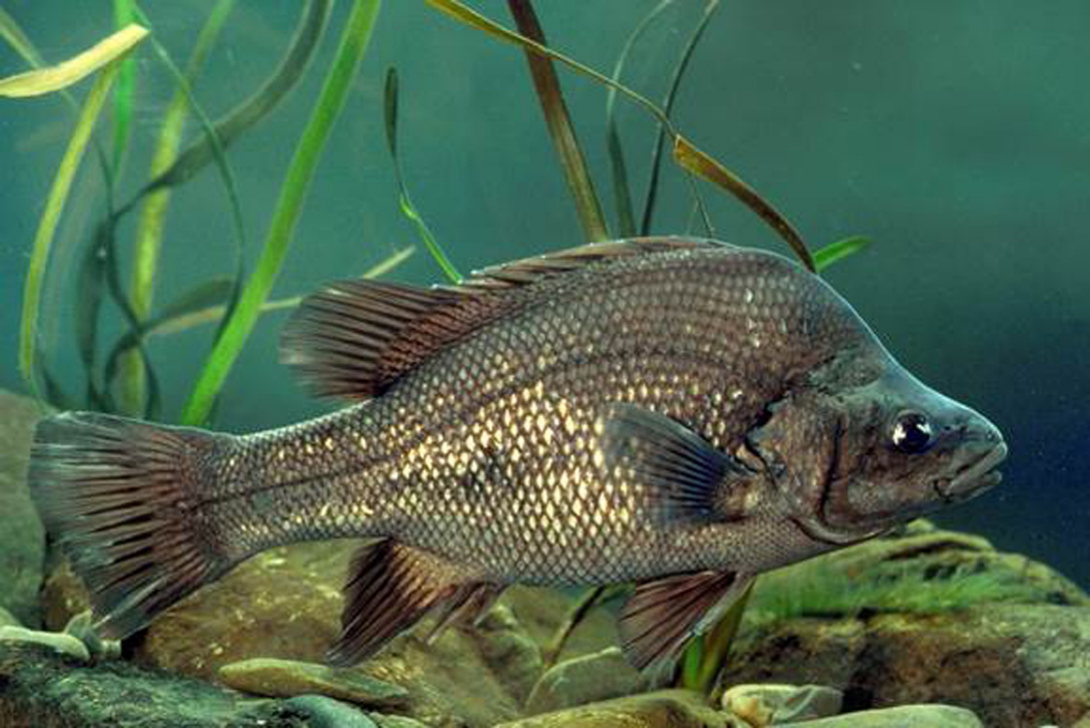
Adult Macquarie Perch (*Macquaria australasica*). Photo by Esther Beaton, ACT Government.

In this study, three eDNA sampling and DNA extraction protocols were tested for the detection of Macquarie Perch (*Macquaria australasica*, Percichthyidae) (Fig. 1), an endangered Australian freshwater fish (Australian Environment Protection and Biodiversity Conservation Act 1999). A pilot laboratory study was conducted comparing these methods and determining the most appropriate sampling and replication strategy based on likely eDNA yield, detection probability and PCR replication for each strategy. The primary objectives of this study were to (1) compare the eDNA yield and detection probability for three sampling and extraction protocols; (2) investigate the effect of three different target amplicon sizes, two marker regions (a nuclear and a mitochondrial gene) and PCR strategy (quantitative PCR and conventional PCR) on detection efficiency; and (3) use the survey effort approach of Wintle et al. (2005) to determine the PCR replication effort required to achieve a specified level of confidence in survey results for each sampling and extraction method using different target amplicon sizes and marker regions.

## Materials and Methods

### Dam experiments

Methods were tested on water samples from a large dam (3 megalitres) at the Narrandera Fisheries Centre (−34.7545° N, 146.54490° E) at Narrandera, New South Wales, Australia. This dam only contains the single fish species *M. australasica*. At the time of sampling, there were 32 adults (600–800 g each) and 351 young of year (50 g each), a total of 36.75–43.150 kg. The dam is drained yearly to collect and count the fish present and this species has high survival but does not breed in the still water environment of the dam. The dam is fenced and netted to prevent access by other vertebrate species. This dam was chosen as it mimics the water conditions of the natural environment in which *M. australasica* are found, has a larger amount of water compared to aquariums, yet the quantity of water and number of fish could still be quantified. At the time of sampling, water conditions in the dam were as follows: water temperature 15.61°C, dissolved oxygen 7.11 mg/L, pH 7.34 and conductivity 0.148 S/m. All laboratory work was conducted in a dedicated eDNA laboratory where DNA extractions took place in a laminar flow cupboard and PCR procedures were conducted in a separate room to DNA extraction in a UV hood. All eDNA extractions and PCR were conducted using aerosol barrier pipette tips and all working surfaces and equipment were wiped down with Lookout DNA Erase (Sigma‐Aldrich, Co. LLC. St. Lewis, MO) before each use. Post‐PCR procedures were conducted in another room in a separate building.

### Evaluating sampling and extraction methods

Three water‐sampling and DNA extraction methods based on the three broad groupings of commonly used capture and extraction methods were compared for multiple water samples collected from the dam (Table 1). Samples of surface water were collected in 1‐L containers (Kartell) after initial rinsing with dam water by submerging just below the surface with a gloved hand. Gloves were changed between samples and the outside of bottles was wiped using Lookout DNA Erase (Sigma‐Aldrich) prior to being transported on ice back to the eDNA laboratory. The outside of the bottle was wiped again before being taken into the eDNA laboratory. A sampling blank was collected randomly using sterile RO water. Three replicate water samples were extracted for each sampling and extraction method. The same 1‐L water sample was used for one replicate of each method.

**Table 1:** Capture and extraction methods used in this study

Method	Capture method	Filter pore size (*μ*m)	Sample volume (mL)	Extraction method	Studies using similar capture and extraction approach
*Filtration and DNeasy*	Filtration	0.45	250	DNeasy Kit	Goldberg et al. (2011, 2013); Pilliod et al. (2013, 2014)
*Filtration and PCI*	Filtration	0.45	250	Phenol‐Chloroform Isoamyl	Barnes et al. (2014); Deiner et al. (2015); Renshaw et al. (2014); Turner et al. (2014a,b)
*Precipitation and DNeasy*	Precipitation		15	DNeasy Kit	Dejean et al. (2011); Ficetola et al. (2008); Piaggio et al. (2014); Thomsen et al. (2012)

The first two eDNA sampling and extraction methods, *Filtration and DNeasy and Filtration and PCI,* used a filtration step to capture eDNA. An amount of 250 mL was pumped through a disposable analytical filter funnel with 47 mm diameter cellulose nitrate filter paper with 0.45 *μ*m pore size (Nalgene) using a peristaltic pump (John Morris). Filters were placed in ethanol, and stored at −20°C until DNA extraction. Filters were removed from the ethanol and air‐dried. Filters were divided in half, one half was extracted using the Qiashredder and Qiagen DNeasy Blood and Tissue extraction method as described in Goldberg et al. (2011) and Pilliod et al. (2013). The other filter half was extracted using method 2, *Filtration and PCI*, a Phenol‐Chloroform Isoamyl DNA extraction as described by Deiner and Altermatt (2014). The third method, *Precipitation and DNeasy*, followed the precipitation and Qiagen DNeasy kit method of Ficetola et al. (2008), adding 15 mL water samples directly to 1.5 mL of 3M sodium acetate and 33 mL absolute ethanol followed by DNA extraction using the Qiagen DNeasy kit (Qiagen GmbH, Hilden, Germany). As the same volume of water used for the two other methods (250 mL) could not be extracted easily using the precipitation method there are issues with direct comparison between all methods. However, generally a filtration method will be able to extract DNA from larger quantities of water than a precipitation method so choice of a precipitation method will generally extract from less water than other eDNA extraction methods. The final elution volume for all samples was 100 *μ*L.

To ascertain whether the above sampling and extraction methods could also be used to successfully detect DNA from *M. australasica* at low densities, bore water from which the dam water was supplied, was spiked with the dam water to provide a 1 in 10 dilution (1:10) and 1 in 100 dilution (1:100) to simulate a biomass of 367–431 g (equivalent to 1 adult or 6–8 young of year) and 36.7–43.1 g (equivalent to 1 young of year) per three megalitres, respectively. As described previously, three replicate samples were extracted and the same 1‐L water sample was used for one replicate of each method. Extraction blanks using the bore water only were also carried out to confirm there was no fish DNA present.

Prior to PCR all samples were quantified using a Qubit fluorometer (Life Technologies, Carlsbad, CA). The instrument was calibrated with the Quant‐iT dsDNA HS Assay (declared assay range between 0.2 and 100 ng; sample starting concentration between 10 pg/*μ*L and 100 ng/*μ*L) following the manufacturer's instructions. For each extraction replicate, 4 *μ*L volumes were measured.

### Effect of target amplicon size and marker region on eDNA detection

On reviewing the literature as at 30 June 2015, primers have been developed to target a product size from 62 bp (Foote et al. 2012) up to 650 bp (Foote et al. 2012; Egan et al. 2013; Deiner et al. 2015) for eDNA detection of individual and multiple species in water samples. The majority of studies target a product less than 150 bp (e.g., Goldberg et al. 2011; Dejean et al. 2012; Takahara et al. 2012; Wilcox et al. 2013). To determine if product size affects eDNA detection, two sets of primer pairs that gave product sizes of 78 bp (12S small) and 390 bp (12S large) were tested to amplify in the 12S region of the *M. australasica* mitochondrial genome (Table 2). This region was selected as it was used in a previous DNA barcoding study of freshwater fish in the Murray‐Darling Basin and all species were uniquely identified using the 12S rRNA gene (Hardy et al. 2011). Universal vertebrate primers already exist that amplify approximately 390 bp in *M. australasica* (Fuller et al. 1998). As the dam is fenced and netted, it is unlikely there is any other vertebrate DNA present except for *M*. *australasica*, the species of interest. In addition, positive detections were sequenced to confirm the detection of *M. australasica* only (see below). A primer pair was designed for a 78 bp fragment using sequences from *M. australasica* (GenBank accession nos. HQ615499–HQ615502). Primer pairs were designed using default parameters in Primer3 version 2.3.4 (Untergasser et al. 2012) (Table 2). As this study used water samples from a dam containing *M. australasica* only, cross‐amplification in other codistributed species was not considered an issue. Primer pair PCR annealing temperatures (Table 2) were optimized using extracted DNA from tissue samples of *M. australasica* (1–2 ng/*μ*L) and eDNA water samples.

**Table 2:** Three primer pairs used in this study describing region, primer sequence, amplicon size in base pairs (bp) and annealing temperature (Anneal.Temp.)

Region	Primer name	Sequence 5′‐3′	Product size (bp)	Taxa	Anneal. Temp. (C)	References
12S rRNA	MT1091L	CAAACTGGGATTAGATACCCCACTAT	390	Universal vertebrate	55	Fuller et al. (1998)
	MT1478H	TGACTGCAGAGGGTGACGGGCGGTGTGT				
12S rRNA	Mac‐aus‐F162^a^	CCGCCTATATACCGCCGT	78	*Macquaria australasica*	55	This study
	Mac‐aus‐R240^a^	CCTGACGTTTTGGGCTGTG				
18S rRNA	Fish_18S_1F	GAATCAGGGTTCGATTCC	271	Universal fish	62	MacDonald et al. (2014)
	Fish_18S_3R	CAACTACGAGCTTTTTAACTGC				

a These primers were not tested on all codistributed species.

The literature review also indicated that mtDNA has been used for species detection in nearly all eDNA studies. There has been only one paper that showed that nuclear DNA (microsatellites) could potentially be amplified (Olsen et al. 2012). In addition to the 12S mtDNA region, a marker in the nuclear 18S rRNA region was also tested in this study using 18S fish primers that amplifies approximately 270 bp in *M. australasica* (MacDonald et al. 2014) (Table 2). This primer pair was selected as it amplifies well in *M. australasica*. This primer pair was also blasted to confirm it only amplifies in fish and positive detections were sequenced to confirm the detection of *M. australasica* only (see below).

### Conventional PCR versus quantitative PCR

Conventional PCR (cPCR) and quantitative PCR (qPCR) were carried out for each sample using the three sets of primers (Table 2). An extraction negative was included with each set of extractions and a PCR negative with each plate of qPCR and cPCR. Six PCR replicates were carried out for each sample and primer pair to give a total of 18 replicates per treatment. For cPCR, a comparison of PCR protocols was carried out in an initial pilot study and the Qiagen Multiplex PCR Master Mix kit performed best. The cPCR was carried out in 20 *μ*L reactions containing 10 *μ*L Qiagen Multiplex PCR Master Mix (Qiagen GmbH), 4 *μ*L Q solution, 0.5 *μ*L of each 10 *μ*m primer, 3 *μ*L of sterile H_2_O and 2 *μ*L extracted DNA. The thermal‐cycling regime was 95°C for 5 min, followed by 45 cycles of 95°C for 30 sec, annealing temperatures (see Table 2) for 30 sec and 72°C for 45 sec and a final extension of 72°C for 5 min. Conventional PCR products were confirmed by gel electrophoresis on a 1.5% agarose gel stained with GelRed (Biotium Inc., Hayward, CA). Positive cPCR products were cleaned using Exo I Nuclease (EXO I) and Shrimp Alkaline Phosphatase (SAP) (Thermo Fisher Scientific Inc., Waltha, MD). EXO I‐SAP reactions were carried out in 10 *μ*L volumes using 0.4 *μ*L EXO I, 1.6 *μ*L SAP and 3 *μ*L double‐distilled water along with 5 *μ*L undiluted PCR product. The thermal‐cycling regime was 15 min at 37°C followed by 15 min at 80°C. Cycle‐sequencing reactions of purified PCR product were carried out using 1 *μ*L BigDye (Applied Biosystems, Foster City, CA), 4.5 *μ*L 5 × Sequencing Buffer, 2.4 *μ*L double‐distilled water, 0.1 *μ*L of the target primer and 1 *μ*L PCR product. Sequencing reactions were purified using a sodium acetate precipitation. DNA pellets were dried before being dissolved in 20 *μ*L HiDi formamide. PCR products were sequenced in both forward and reverse directions using dideoxy chain termination chemistry with Big Dye v3.1 following recommended ABI protocols and run on an ABI3100 automated capillary sequencer (Applied Biosystems). Forward and reverse sequences were aligned using Geneious 8.1.7 (Kearse et al. 2012). Consensus sequences were then aligned to sequences used for primer design (Table 2) to confirm the amplified product matched that of the targeted region. The number of positives was determined by the number of replicates that produced bands of expected size and provided a *M. australasica* sequence.

The same primer pairs were analyzed using qPCR on the LightCycler^®^480. Each reaction was made up to 20 *μ*L, containing 10 *μ*L of Power SYBR Green Master mix (Applied Biosystems), 0.5 *μ*L of each 10 *μ*m primer, 7 *μ*L of sterile H_2_O and 2 *μ*L of DNA extract. The thermal‐cycling regime was as described above for cPCR as well as a melt curve step up to 95°C. The number of positives from the six replicates was determined for each sample. Testing negative was indicated by no exponential phase at any point during the 45 cycles. To confirm assay specificity, the product of positive qPCR reactions were sequenced as described above for cPCR. Standard curves were constructed from whole genomic DNA extracted from fin clips of *M. australasica* from the hatchery and diluted to 0.001, 0.01, 0.1 and 1 ng as described by Pilliod et al. (2013). The DNA concentration was quantified in the standard samples using a Qubit fluorometer (Life Technologies) as described previously. To determine if inhibition was present during qPCR, differences in the quantification cycle (Cq) between the undiluted samples (1) and diluted (1:10) samples were evaluated (see Gibson et al. 2012).

### Analysis of the relationship between eDNA concentration and biomass

I initially examined the relationship between eDNA sample concentration (Qubit measured) and biomass estimated from the undiluted and diluted samples to determine if there was a correlation between the dilutions/biomass and the eDNA yield for each extraction method. I then examined the relationship between eDNA qPCR concentration and estimated biomass for each extraction method and amplicon. Biomass was estimated for the undiluted and diluted samples using the lowest likely weight of 36.75 kg in the three megalitre dam. A Type II regression was carried out for both analyses and statistical analyses were carried out using IBM SPSS Statistics (version 23; SPSS Japan Inc., Tokyo, Japan).

### Analysis of eDNA detection rates

Generalized linear models (GLMs) were used to analyze detection rates of *M. australasica* in response to the three water sampling and DNA extraction methods, amplicon size and dilution factor. For analyzing detection and nondetection rates, a binomial distribution was used with *P*‐values based on Chi squared statistics. A comparison of cPCR and qPCR detection rates was carried out initially with no other effects. As PCR strategy had an effect on detection rates, data from qPCR and cPCR assays were analyzed separately. I fitted a full model for detection rates for qPCR and cPCR separately with effects of method, dilution factor, amplicon size and replicate (PCR~Method+Dilution.factor+Amplicon.size+Replicate). Interactions were not included so as not to overparameterize the model. All statistical analyses for GLM were carried out using Firth's bias reduction logistic regression (Firth 1993) in the *logistf* R package (Heinze et al. 2013) in the program R, version 3.0.1 (Team 2013).

### Estimating detection probabilities to determine PCR replication strategy

To determine the PCR replication effort required to achieve a threshold probability of detection of *M. australasica*, given that it is present in the sample (*D *=* *0.95 for this study), I used the survey effort equation:

$$D=\text{Pr}\left(\text{detected}|\text{present}\right)=1-{\left(1-p\right)}^{n},$$

where *p* is the single‐visit detection probability of the species, given presence, and *n* is the number of repeat visits (Wintle et al. 2005). As described by Wintle et al. (2005), as survey effort increases, the probability of detecting the species when it is present will also increase. The probability of detection was determined over the 18 PCR replicates (three water samples × 6 PCR replicates) for each method and for each of the three amplicons for the 1 in 100 dilution (which best imitates the low DNA quantity likely to be encountered in a field situation).

## Results

For this study, all collection negative controls, extraction negative controls and PCR negative controls tested negative with no evidence of contamination. The standard curve error ranged from 0.040 to 0.0150, efficiency ranged from 91% to 97%, slope ranged from −3.385 to −3.898 and Y intercept ranged from 35.48 to 41.68 cycles. Positive detections were always confirmed with the expected *M. australasica* sequence. The Cq value (quantification cycle value) between diluted (1:10) and undiluted (1) samples was not significant suggesting no or limited effect of inhibition.

### Evaluating sampling and extraction methods

**Figure 2:**
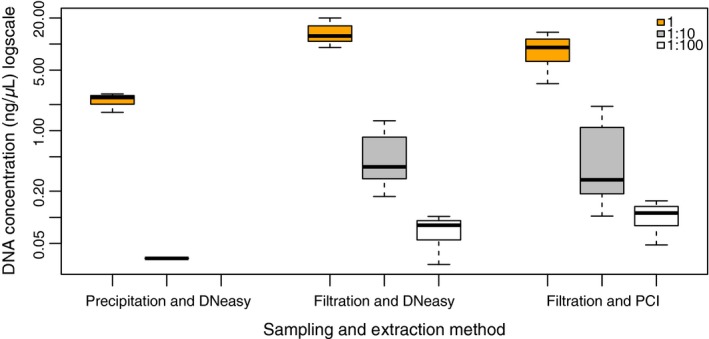
Comparison of DNA recovered from each sampling and extraction method with no dilution (1), 1 in 10 dilution (1:10) and 1 in 100 dilution (1:100) showing the mean and standard error of measurable DNA (ng/*μ*L) (on a log scale) for three water sample replicates.

Except for the 1 in 100 dilution for the *Precipitation and DNeasy* method, all three methods had quantifiable DNA at each dilution (Table S1, Fig. 2). The two filtration methods (*Filtration and DNeasy* and *Filtration and PCI*) had higher yields of measurable DNA than the *Precipitation and DNeasy* method (Table S1, Fig. 2). The amount of quantifiable DNA for each dilution (1:10 and 1:100) was not consistent with an expected tenfold or 100‐fold decrease from the concentration measured in the undiluted samples (1) (Table S1).

### Effect of PCR strategy, target amplicon size, dilution and marker region on eDNA detection

**Figure 3:**
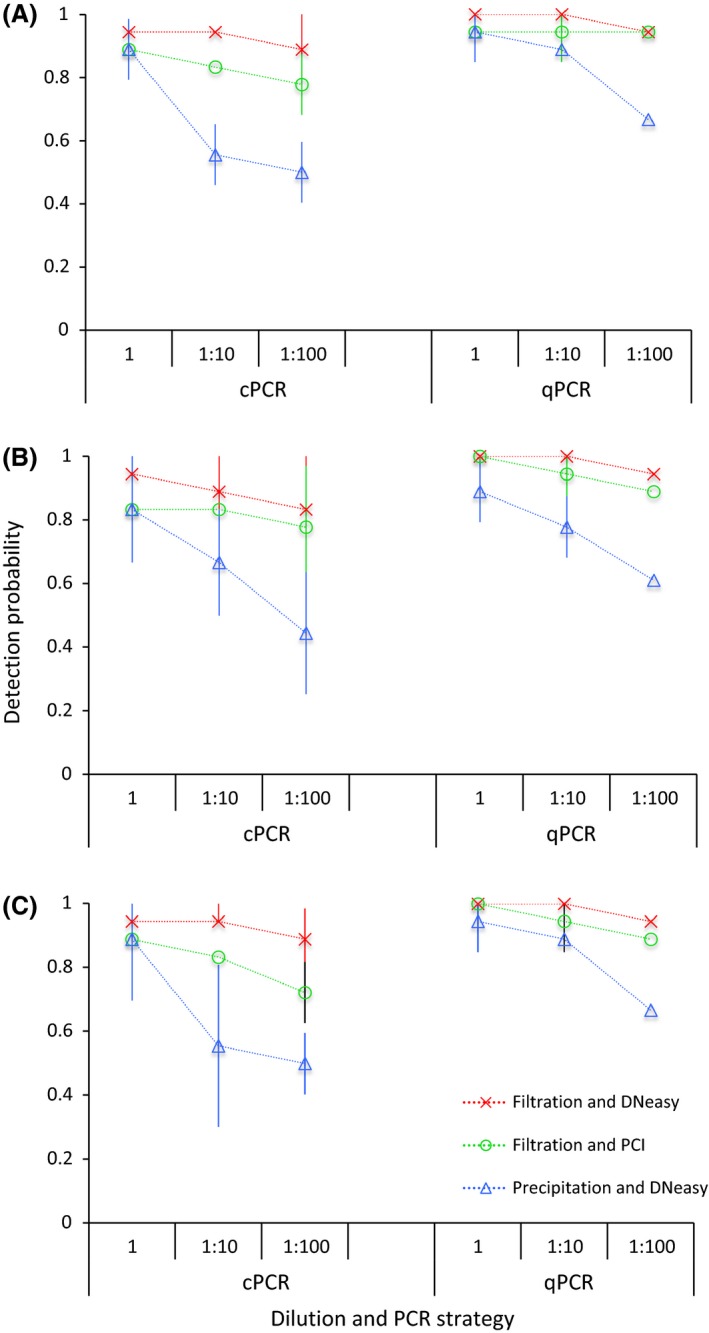
Detection probability for *M. australasica* water samples for each sampling and extraction method based on the mean and standard error for 18 replicates (six PCR replicates × 3 water samples), dilution factor (1, 1:10, 1:100), PCR strategy (qPCR and cPCR), and target amplicon (A 12S small, B 18S and C 12S large).

All three sampling and extraction methods yielded eDNA from which *M. australasica* was detected using the three target amplicons, but detection rates depended on the sampling and extraction protocol used (Fig. 3). The choice of PCR strategy (qPCR or cPCR) had a significant effect on detection probability (Table S3). Overall detection rate across all extraction methods, target amplicons and dilutions for qPCR was 0.911 compared to 0.800 for cPCR. The best GLM models for cPCR included an effect of DNA extraction method and dilution factor (Table S4). The models of the qPCR data revealed effects of sampling and extraction method, but no effect of dilution factor (among the range of dilution factors evaluated) (Table S5). These models identified a significantly lower detection rate of the *Precipitation and DNeasy* method compared to the *Filtration and DNeasy* method (Tables S4 and S5, Fig. 3). The overall cPCR and qPCR detection rates for *Filtration and DNeasy* was 0.913 and 0.981, 0.821 and 0.944 for *Filtration and PCI* and 0.654 and 0.808 for *Precipitation and DNeasy*. The detection probability decreased as the dilution factor increased for all three methods and amplicon targets (Fig. 3). There was no significant effect of target amplicon size or marker region on detection rates using either PCR method (Tables S4 and S5).

### Relationship between biomass and eDNA concentration

**Figure 4:**
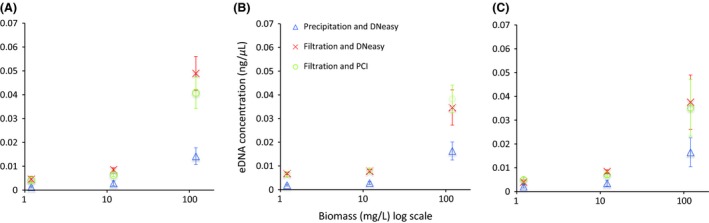
Relationship between eDNA concentration and *M. australasica* biomass/dilution per 1‐L water (on a log scale) showing the mean and standard error for each sampling and extraction method and target amplicon (A 12S small, B 18S, and C 12S large).

The correlation between the dilution/biomass of perch and eDNA sample concentration (Qubit) was significantly positive for all methods (*Precipitation and DNeasy*:* y* = 0.0195*x* − 0.1183, *R* ^2^ = 0.939, *P *<* *0.001, *Filtration and DNeasy*:* y* = 0.1185*x* − 0.4199, *R* ^2^ = 0.85027, *P *<* *0.001, *Filtration and PCI*:* y* = 0.0195*x*−0.1183, *R* ^2^ = 0.939, *P *=* *0.004). There was positive and significant correlation between the estimated perch biomass at each dilution and the eDNA qPCR concentration for all extraction methods and amplicons (Table 3; Fig. 4).

**Table 3:** Results of correlation between biomass and eDNA concentration from qPCR using Type II regression for each sampling and extraction method and target amplicon

Method	Amplicon	*y*	*R* ^2^	*P*
Filtration and DNeasy	12Ssmall	0.0004x + 0.0041	0.958	0.000
	18S	0.0003x + 0.0046	0.943	0.000
	12Slarge	0.0003x + 0.0054	0.947	0.000
Filtration and PCI	12Ssmall	0.0003x + 0.0032	0.949	0.000
	18S	0.0002x + 0.0046	0.937	0.000
	12Slarge	0.0002x + 0.0052	0.900	0.000
Precipitation and DNeasy	12Ssmall	0.0001x + 0.0014	0.878	0.000
	18S	0.0001x + 0.0023	0.812	0.000
	12Slarge	0.0001x + 0.0016	0.895	0.000

### Calculating detection probabilities and required survey effort

**Figure 5:**
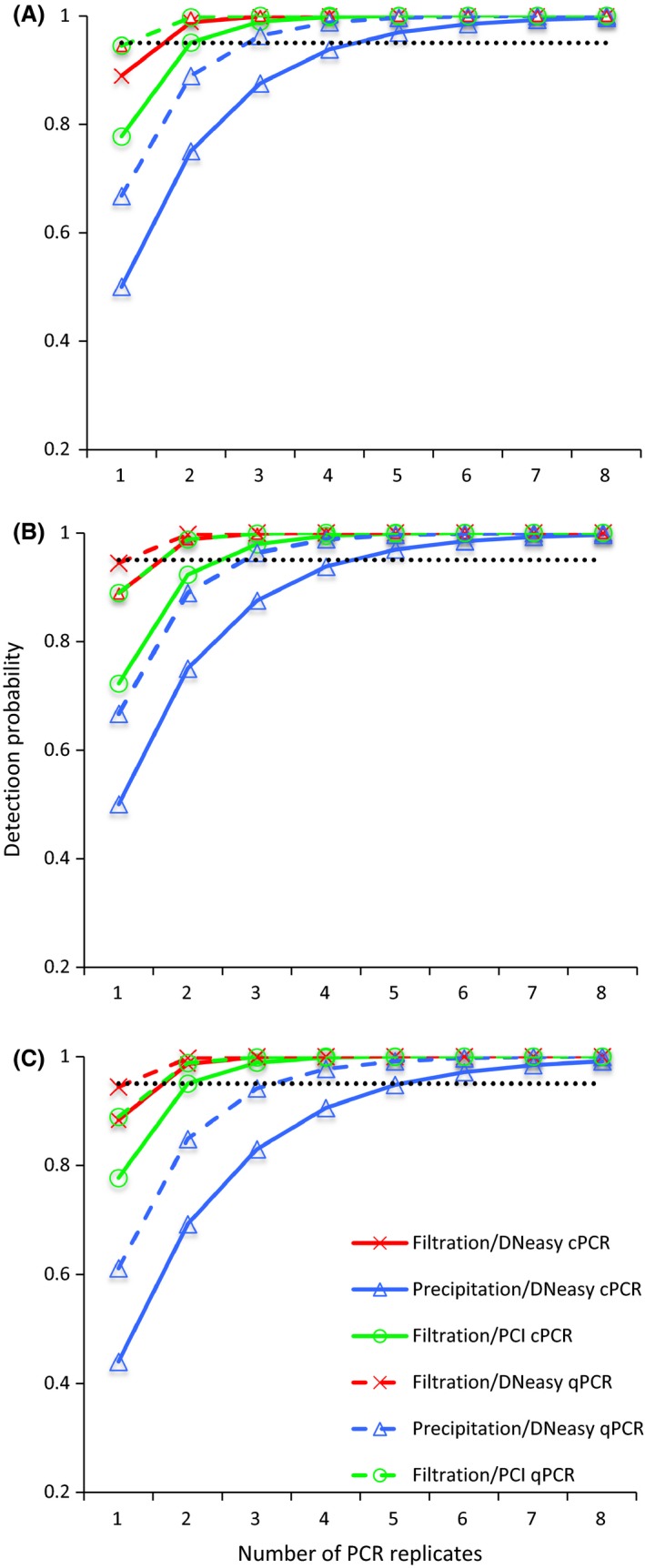
The PCR replication effort required to detect the presence of *M*. *australasica* in 1:100 dilution water samples with probability (*D*) of 0.95 (dotted line) comparing PCR strategy and target amplicon (A 12S small, B 12S large and C 18S) for each sampling and extraction method.

The PCR replication effort required to detect the presence of *M*. *australasica* in 1:100 dilution water samples with probability (*D*) of 0.95 ranged from two to six replicates (Fig. 5). More replicates were required for the *Precipitation and DNeasy* method (range 3–6) compared to *Filtration and DNeasy* and *Filtration and PCI* (range 2–3). More replicates were required for cPCR (range 2–6) compared to qPCR (range 2–4). Variation in the number of replicates required to achieve 0.95 detection probability was slightly higher for the 18S marker (range 2–6) compared to the 12S markers (range 2–5).

## Discussion

This study has demonstrated that all three sampling and extraction methods were able to detect *M. australasica* at different dilutions and with differences in detection probability. These differences could be mitigated by determining the appropriate PCR replication strategy. Choice of sampling and extraction method and PCR strategy, rather than amplicon size and marker region, had the biggest effect on detection probability and PCR replication. Choice of sampling and extraction methods for eDNA studies is dependent on cost, sampling location, species and ecosystem differences and preference. Ideally, a method that maximizes eDNA yield and detection will be used but to date, no single method is being used across eDNA studies. Evaluating the effect a sampling and extraction method will have on the detection probability and determining the survey effort or PCR replication required to maximize detection and minimize false positives and negatives is a useful first step for eDNA occupancy studies.

### Choice of water sampling and DNA extraction method

In this study, a filtering step to capture eDNA followed by either extraction by the Qiagen DNeasy kit or PCI performed better than the *Precipitation and DNeasy* method in relation to eDNA yield, detection probability and PCR replication effort for *M. australasica*. All methods had detectable eDNA but there were significant differences in eDNA yield, detection probabilities and PCR replication effort to detect *M. australasica* eDNA. Higher eDNA yield does appear to correspond to higher detection probability. One important factor contributing to this result was the quantity of water analyzed. The two filtration methods used a volume of 250 mL compared to the precipitation method that used only 15 mL. However, Piaggio et al. (2014) found that a sodium acetate precipitation method performed better than a filtration approach even though the precipitation method isolated DNA from the least amount of water (15 mL vs. 2‐L). Multiple protocols may also maximize detection accuracy as demonstrated by Deiner et al. (2015) who found precipitation and the PowerWater kit to be optimal for eubacteria metabarcoding and filtration and the Qiagen DNeasy kit optimal for eukaryote detection using the same amount of water.

There are advantages and disadvantages to any method used. As discussed in Renshaw et al. (2014), extraction using PCI relies on harmful chemicals but can be a cheaper option than those incorporating commercial extraction kits. There could also be a potentially increased risk of contamination for sampling and extraction methods requiring multiple steps such as filtering and removing filters for extraction. The *Precipitation and DNeasy* method is likely to require a greater survey effort such as PCR replication and sampling as seen in this study and others (Dejean et al. 2012; Ficetola et al. 2015), but it may be more suitable for remote sampling or collection of large numbers of water samples from sites. Although other methods are likely to have higher eDNA yield and detection rates than the *Precipitation and DNeasy* method, increasing the survey effort (via increased PCR replicates or more sampling) could compensate for these differences when this sampling/extraction method has other advantages.

### Effect of amplicon size, marker region and PCR strategy

Although smaller amplicons are expected to amplify more successfully in degraded DNA samples (Deagle et al. 2006), amplicon size and marker region were not limiting factors for detecting *M. australasica* eDNA in this study. The study system used here was a closed system (dam) with a higher density of fish than would be seen in a field environment. Smaller amplicons may perform better than larger ones in a river or stream environment, but no effect of amplicon size was observed over serial dilutions in this study. A caveat associated with this result is that, by necessity, primer design also differs between the amplicons of different sizes, and this may contribute to the observed results (i.e., the larger amplicon may have simply had better primers than the smaller amplicon). However, it is notable that several other studies have also used large amplicons successfully to detect species, including invertebrates, using eDNA (e.g., Egan et al. 2013; Deiner and Altermatt 2014; Deiner et al. 2015). The finding in this study that amplicon size was not limiting, corresponds to the findings of two studies that the most abundant eDNA particles are 1–10 μm across, suggesting capture of individual cells or mitochondria (Turner et al. 2014a; Wilcox et al. 2015).

Nuclear DNA is expected to be in lower concentrations than mtDNA in environmental samples as compared to nuclear DNA, mtDNA is present in cells in greater magnitude (Birky et al. 1989). This was not the case for this study, although the nuclear 18S amplicon did require a slightly higher survey effort compared to the two mtDNA amplicons to achieve a 0.95 detection probability. One previous study by Olsen et al. (2012) amplified nuclear DNA (microsatellites) for hellbender eDNA but not as successfully as a mtDNA marker. This would need to be pursued further in laboratory and field experiments but is a promising indicator that amplification of nuclear eDNA is possible from water samples. Nuclear DNA markers for eDNA detection of fish may be useful as alternative or additional markers for species identification and potentially to delineate species boundaries and detect hybridization, which mtDNA markers may be unable to do for some fish taxa (Ward et al. 2009; Hardy et al. 2011).

As shown in previous studies, qPCR is a potentially more effective method for species detection than cPCR (Turner et al. 2014b). Using a cPCR strategy compared to qPCR in this study resulted in a significantly lower detection rate for all methods and required more survey replicates. Choosing a cPCR approach may therefore require a greater PCR replication effort to achieve a given species detection probability, particularly when combined with a sampling and extraction method that results in a low eDNA yield. The other advantage to a qPCR approach is the potential to estimate abundance/biomass from eDNA concentrations in water samples (Takahara et al. 2012; Turner et al. 2014b).

In this study, there was a positive correlation between the biomass of *M*. *australasica* and eDNA sample concentration and perch biomass and eDNA concentration as measured via qPCR. This was positive and significant for all methods and amplicons. These results are consistent with the findings of other studies on biomass of carp (Takahara et al. 2012) and tailed frogs (Pilliod et al. 2013). Although there were differences in the eDNA concentrations between methods, each method individually showed a positive linear relationship suggesting that choice of method was not limiting for assessing biomass/abundance. As this was a closed dam with a high density of fish and only three biomass data points based on the dilutions, it is difficult to determine how applicable these results are for estimating biomass of *M. australasica* in a field situation. Pilliod et al. (2013) found that their field surveys included eDNA samples with very high and very low eDNA concentrations resulting in statistical outliers. Increasing the replication is likely to reduce the impact of this potential problem (Pilliod et al. 2013) but also suggests that further studies are required to determine the accuracy of estimating abundance from field eDNA samples.

### Use of DNA extraction and PCR replication to account for variation in detection probability among methods

Current statistical methods for detection and occupancy studies can be used effectively for eDNA studies as these statistical approaches account for imperfect detection and a multiple sampling strategy will comply with statistical assumptions (Schmidt et al. 2013). The survey effort approach of Wintle et al. (2005) used in this study provides an effective way of assessing the PCR replication effort required, based on the predetermined detection probability, for a chosen sampling and extraction approach. In addition, this approach can also be used to assess the survey effort required to be confident that the species is absent (Garrard et al. 2015). Determining an appropriate level of replication for a study needs to take into account variation in detection probabilities between species and within species in different ecosystems.

In this study, the PCR replication effort ranged from two to six replicates depending on the method, target amplicon size and marker region. The number of PCR replicates per eDNA sample for single species detection has ranged from two to fifteen (Ficetola et al. 2008; Dejean et al. 2012). As discussed in Ficetola et al. (2015) the number of replicates for eDNA samples needs to be balanced between reducing the risk of missing taxa and potentially increasing the risk of false positives as well as cost and workload. The survey effort method used here to estimate PCR replication could also be used to assess the number of samples per waterbody. The number of PCR replicates can be estimated from a likely detection probability rather than based on guesswork or other researchers' protocols, which could lead to too many replicates inflating the workload and cost or too few replicates, which could reduce the reliability of results.

As has been previously recommended for eDNA and noninvasive studies, pilot studies for a particular species or ecosystem are essential for optimizing sampling and extraction methods, maximizing DNA recovery, assessing error rates and determining sample and PCR replication strategies (Piggott and Taylor 2003; Deiner et al. 2015; Ficetola et al. 2015). The framework provided in this study is a useful step prior to embarking on a full‐scale occupancy study to determine eDNA yield, the potential detection probability and survey effort required for the choice of sampling and extraction method and laboratory approach. Incorporating statistical methods to determine detection probability and survey effort will improve the robustness of occupancy models and inferences on the presence and absence of aquatic fauna for biodiversity assessment in macrobial eDNA studies.

## Data Accessibility

All data are available in the Supporting Information.

## Conflict of Interest

None declared.

## Supporting information


**Table S1.** Concentration of eDNA with no dilution (1), 1 in to dilution (1:10) and 1 in 100 dilution (1:100) for three capture and extraction methods.
**Table S2.** Detection data, qPCR concentration and sequence data for each replicate for three capture and extraction methods.
**Table S3.** Results of generalized linear models (GLM), explaining differences in detection rates as a result of PCR strategy.
**Table S4.** Results of generalized linear models (GLM), with the best model to explain the variation in detection success for cPCR with effects of extraction method, dilution factor, amplicon size and replicate.
**Table S5.** Results of generalized linear models (GLM), with the best model to explain the variation in detection success for qPCR with effects of extraction method, dilution factor, amplicon size and replicate.

**Appendix S1.** R Script for GLM analyses.
