# Supplementary material for: Evaluating the effects of laboratory protocols on eDNA detection probability for an endangered freshwater fish
Source: Ecol Evol. 2016 Mar 17;6(9):2739–50. doi: 10.1002/ece3.2083 (PMC4798829; doi:10.1002/ece3.2083)
Supplement: Supplementary file 2 — Appendix S1. R Script for GLM analyses. [file ECE3-6-2739-s002.docx]

#R Script for GLM analyses

# GLM to analyse detection rates as a result of different water sampling and extraction methods, amplicon size, dilution factor and replicate

#first change directory to where your csv file is

#read the file

edna.data.bin<-read.csv("GLMedna.binary.csv", sep=",", header=TRUE)

head(edna.data.bin)

attach(edna.data.bin)

Dilution.factor<-as.factor(Dilution)

library(logistf)

#Used Firths bias reduction logistic regression

#between PCR only

lr.PCR.detect<-logistf(Detect~PCR)

summary(lr.PCR.detect)

#cPCR response variable

#full model is effects of 4 major variables

lr.full.cPCR<-logistf(cPCR~Method+Dilution.factor+Amplicon.size+Replicate)

summary(lr.full.cPCR)

#'qPCR response variable

#full model is effects of 4 major variables

lr.full.qPCR<-logistf(qPCR~Method+Dilution.factor+Amplicon.size+Replicate)

summary(lr.full.qPCR)
